# Effects of Stellate Ganglion Block on Healing of Fractures Induced in Rats

**DOI:** 10.1155/2020/4503463

**Published:** 2020-08-16

**Authors:** Hasan Kizilay, Husamettin Cakici, Erkan Kilinc, Tulin Firat, Tolgahan Kuru, A. Alper Sahin

**Affiliations:** ^1^Department of Orthopedics and Traumatology, Gerede Public Hospital, Bolu, Turkey; ^2^Department of Orthopedics and Traumatology, Abant Izzet Baysal University Medical Faculty, Bolu, Turkey; ^3^Department of Physiology, Abant Izzet Baysal University Medical Faculty, Bolu, Turkey; ^4^Department of Histology and Embryology, Abant Izzet Baysal University Medical Faculty, Bolu, Turkey; ^5^Department of Orthopedics and Traumatology, Onsekiz Mart University Medical Faculty, Canakkale, Turkey; ^6^Department of Orthopedics and Traumatology, Erbaa Public Hospital, Tokat, Turkey

## Abstract

**Objective:**

Sympathetic blocks are used as an adjunct for pain management in the treatment of orthopedic and traumatic conditions. Stellate ganglion (ganglion stellatum) provides sympathetic innervation of the head, neck and cervicothoracic regions, and upper extremities. No study was found in the literature investigating the effects of stellate ganglion block performed in the upper extremity, on blood supply to bone, density, vascularization, and bone metabolism. Therefore, the objective of this study was to investigate the effects of stellate ganglion block on healing of closed forearm fractures that were induced in rats. *Material and Methods*. A total of 42 Wistar albino rats weighing between 398 and 510 g were used in this study. The rats were randomly divided into 2 groups with one group treated with stellate ganglion and the other included as the control group. In each 2 groups, a closed forearm fracture was created, confirmed with X-ray, and then stabilized by splint application. The forearm bones were examined with X-ray views on the same day and were then decalcified.

**Results:**

When histological findings of the fracture region were examined, predominantly cartilage and less woven bone were found in 7 rats, equally distributed cartilage and immature bone in 14 rats, and predominantly imitation bone and less cartilage formation in 21 rats. In the control group, the agreement between the 1st and 2nd orthopedists for the radiological evaluation of bone formation was moderate.

**Conclusion:**

The group administered stellate ganglion block showed a more significant fracture healing.

## 1. Introduction

Fractures are one of the most commonly encountered types of injury in orthopedics and traumatology practice, and surgical intervention is performed in many of such injuries.

According to the statistics of the United States of America (USA), 590,193 persons experience upper extremity fractures each year, with most common ones being distal radius and ulnar fractures followed by hand bone fractures, proximal humerus fractures, and clavicular fractures [1].

Fracture healing is a specific wound healing response occurring with new bone formation. The mechanism of fracture healing is a complex biological process, which follows specific regenerative patterns [2]. Minimal pain feeling and comfort of patients with surgical procedures are important in fracture healing. Sympathetic blocks are the methods used as an adjunct for pain management in the treatment of orthopedic and traumatic conditions [3].

Selective block of the sympathetic trunk was described for the first time by Sellheim, and later by Kappis in 1923, and Brumm and Mandl in 1924 [4]. This block is effectively used by algologists in several diseases such as reflex sympathetic dystrophy, herpes zoster, postherpetic neuralgia, phantom pain, Paget's disease, neoplasms, radiation neuritis, central nervous system-induced pain, and persistent angina pectoris, as well as in vascular disorders such as Raynaud's syndrome, freeze, vasospasm, obstructive and embolic vessel diseases, and scleroderma [5].

Stellate ganglion (ganglion stellatum) provides sympathetic innervation of the head, neck and cervicothoracic regions, and upper extremities. Blockage or removal of the stellate ganglion results in vasodilation in these regions, which in turn leads to increased blood flow into these regions. In an experimental study, it was found that vasoconstriction occurred in the bacillary and middle cerebral arteries of rats with induced subarachnoid hemorrhage, while cervical sympathectomy (stellate ganglion block) decreased serum concentration of endothelin 1, a vasoconstrictor agent, and increased the concentration of calcitonin gene-related peptide (CGRP), a vasodilator [6].

In the literature screening, we could not find a study investigating the effects of stellate ganglion block applied in the upper extremity, on blood supply to bone, density, vascularization, and bone metabolism. Therefore, the objective of this study was to investigate effects of stellate ganglion block on healing of a closed forearm fracture induced in rats.

## 2. Material and Methods

Before the beginning of the study, the necessary approval was received from the Experimental Animals Researches Ethics Committee of our hospital. The study was conducted in compliance with the principles of *Guide for the Care and Use of Laboratory Animals*. A total of 42 12-month-old male Wistar albino rats weighing between 398 and 510 g were obtained from the Experimental Animals Researches Laboratory and were used in this study. Rats were kept at a light/dark cycle (10-14 hours), at normal room temperature and humidity, and were fed with standard pellet feed and tap water during the experiments. The rats were divided into two groups as follows:

*Group 1 (controls)*: the group without stellate ganglion application and with forearm fracture induced (*n* = 20).

*Group 2 (experiment)*: the group with stellate ganglion application and forearm fracture induced (*n* = 22).

The rats were anesthetized with 90 mg/kg ketamine hydrochloride (Ketalar, Eczacibasi) and xylazine chloride (Rompun, Bayer). A left jugular incision was made in Group 1 rats, and the incision lines were sutured without excision of the stellate ganglion. Group 2 rats underwent excision of the stellate ganglion through a left jugular incision bypassing the skin, subcutaneous tissues, and muscular structures.

The operation sites were sutured and closed following excision of the stellate ganglion. A four-point principle was then applied, and a forearm two-fracture model was produced in the left arms of the subjects. Formation of the fracture was radiographically confirmed.

Group 1 (control group) was not given any agent in the postoperative period and followed up until sacrifice at the end of the 6th week, whereas rats in Group 2 (experiment group) were administered a single dose of 1 cc/g Parol vial (Bayer) a day for postoperative analgesia.

At the end of the 6th week, all rats were sacrificed by cervical dislocation under general anesthesia. Left forearms of the rats were disarticulated from the elbow and wrist joints and were amputated. The pieces obtained were kept in 10% formaldehyde solution until the analysis.

### 2.1. Radiological Evaluation

Anteroposterior and lateral X-rays were obtained in all subjects at the end of the 6th week. Imaging was performed with a high-resolution digital radiography system (Siemens, Multix C, Japan). The imaging was standardized using 66 kV, 1.82 msn, 1.20 mAs, and ×1 magnification from a distance of 110 cm. The evaluation was performed with a radiologist and an orthopedist who were not aware of the study using the Lane and Sandhu Scoring System [7].

### 2.2. Histopathological Evaluation

For histopathologic examination, bone tissue samples involving the fracture union site in the left forearm were fixed in 10% buffered neutral formaldehyde solution. Bone tissue samples were then prepared by 10% buffered formalin and mixed with 20% formic acid (UN 1779, Merck, Darmstadt, Germany). The mixture was kept in acid solution for 24 hours and decalcified. Following decalcification, bone tissue was cut so as to center the osteotomy field and divided into two parts in the sagittal plane. The pieces obtained were kept 24 minutes each in 70%, 80%, and 96% ethyl alcohol series and then in four different acetone series. The samples were then subjected to two different xylene series for 30 minutes for dehydration. Following immersion two times with paraffin for one hour each, the tissue samples were then embedded into paraffin blocks. Sections of 4-6 *μ* were cut from the paraffin blocks and stained with hematoxylin-eosin (H&E), nuclear fast red, and Alcian blue and were evaluated under a Nikon Optiphot-2 light microscope according to the histologic scoring system described by Huo et al. [8].

### 2.3. Statistical Analysis

Radiologic and histopathologic evaluations obtained from the tissue samples taken from the operated rats were analyzed using SPSS 17.0 Windows software (SPSS Inc., Chicago, IL, USA). Data are presented as numbers (*n*) and percentages (%). Nominal variables were compared with each other utilizing the Chi-squared and Fisher exact tests. Agreements between the observers were examined with Kappa analysis. *p* < 0.05 values were considered statistically significant.

## 3. Results

**Figure 1:**
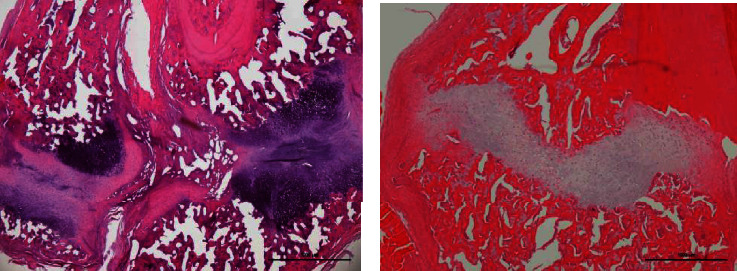
Predominantly cartilage tissue and less woven bone trabeculae between fracture ends in the sections stained with H&E.

The study included a total of 42 rats with 22 being in the experiment and 20 in the control group. When histopathologic findings of the fracture regions were compared between the groups, the rate of predominantly immature bone (woven) and less cartilage was significantly higher in the experimental group compared to the control group (*p* = 0.006) (Table 1).  Figure 1 shows predominantly cartilage tissue and less woven bone trabeculae between fracture ends in the sections stained with H&E.

Results of the first observer for bone formation, union, and remodeling after evaluation in the experimental and control groups are given in Table 2. Results of the second observer for bone formation, union, and remodeling after evaluation in the experimental and control groups are given in Table 3.

Accordingly, when the results of the first observer for bone formation, union, and remodeling were compared between the experimental and control groups, no statistically significant difference was observed between the groups (for all *p* > 0.05). When results of the second observer for bone formation, union, and remodeling were compared between the experimental and control groups, again no statistically significant difference was observed between the groups (for all *p* > 0.05).

When the tables were examined, there was a moderate agreement between the first and second observers in terms of the results of bone formation in the control group (*ƙ* = 0.478) (*p* = 0.001). Again, there was a moderate agreement between the first and second observers in terms of the results of bone formation in the control group (*ƙ* = 0.434) (*p* = 0.004).

**Figure 2:**
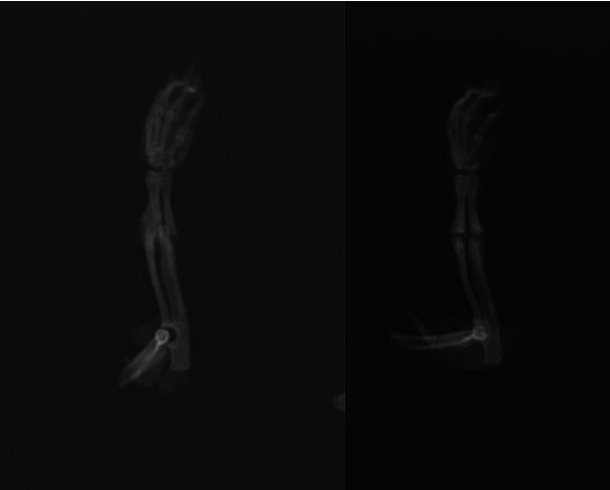
X-ray images of the control group at the end of the sixth week.

**Figure 3:**
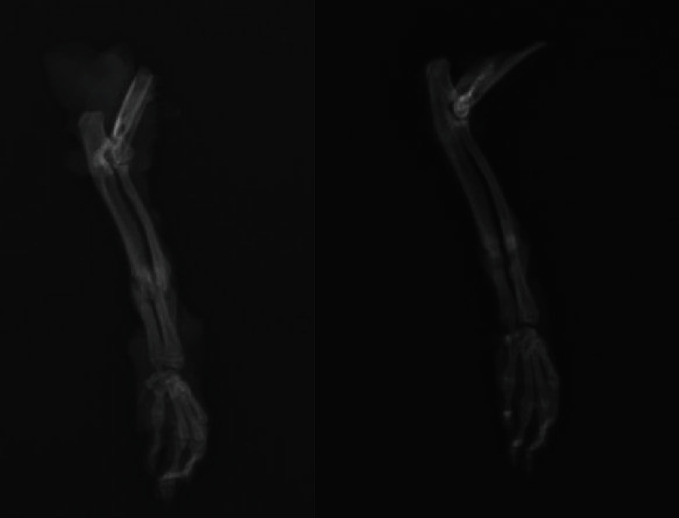
X-ray images of the experimental group at the end of the sixth week.

X-ray images of the experimental and control groups at the end of the sixth week are seen in Figures 2 and 3.

There was a low-to-moderate level of agreement between the first and second observers in terms of the bone union results in the control group (*ƙ* = 0.302) (*p* = 0.007).

There was no significant agreement between the first and second observers in terms of the results of bone union in the experimental group (*p* > 0.05). A low-to-moderate agreement was found between the first and second observers in terms of the results of remodeling in the control group (*ƙ* = 0.381) (*p* < 0.001).

A low-to-moderate agreement was found between the first and second observers in terms of the results of remodeling in the experimental group (*ƙ* = 0.432) (*p* < 0.001).

## 4. Discussion

The objective of this experimental study was to investigate the effect of stellate ganglion block on a closed fracture model induced in forearms of rats and to discuss the results with the current literature. In addition, agreement between the orthopedists in terms of radiologic evaluation of callus formation, onset of bone union, and fracture line was also examined.

According to our results, the rate of predominantly immature bone was higher and the rate of cartilage was lower in the stellate ganglion block group, while predominantly cartilage and less immature bones were found in the control group. There was a moderate agreement between the orthopedists.

Bone fracture is a complex process, influenced by many clinical, mechanical, biological, biochemical, molecular, and neurohumoral factors, and there are a lot of clinical and experimental studies performed on this subject [2, 9–15]. In general, numerous factors negatively affect fracture healing including high-energy traumas, complete fractures, accompanying vascular problems, multisegmental fractures, open fractures, failed reduction, failure in good fixation, advanced age, fractures on the articular surface, nutrition, systemic diseases (diabetes mellitus, malignancies, infections, anemia, and endocrinologic disorders), smoking, and drugs used [2, 9–13, 16]. On the other hand, some physiotherapy agents (magnetic field stimulation, therapeutic ultrasonography, and electrotherapies), growth hormone factors, and prostaglandins have been reported to positively affect fracture healing [2, 14, 15]. In the present study, we standardized the groups as much as possible in order to minimize or rule out other effects that might occur on the fractures. Gender- and age-matched healthy rats were used, and the same fracture model was created in all rats. The rats were subjected to a 12-hour light/dark cycle in order to provide physiological circadian rhythms. An environmental temperature of 20-24°C was provided in order to keep cage temperatures at an optimal level. All rats were fed with the same pellet feed and given feed and water *ad libitum*. In addition, the fracture line was confirmed with radiologic images, and appropriate immobilization was provided in order to evaluate and improve the fracture. We believe that our results are reliable as features of both experimental and control groups were similar.

In our study, the rats were sacrificed at the end of the 6th week. Evaluating the main histologic phases, predominantly cartilage and less immature (woven) bone tissues were observed in 6 rats in the control group and in one rat in the experimental group. In addition, rates of immature cartilage and immature bone were similar in the total of 14 rats with 9 being in the control and 5 in the experimental group, whereas predominantly immature bone and less cartilage were seen in 21 rats with five rats being in the control group and 16 in the experimental group. In addition, callus formation, onset of bone union, invisible fracture line, and complete union phases were radiologically observed. In general, looking at the radiologic and pathologic phases, our results are consistent with each other. We did not observe the inflammation phase pathologically, which is seen between the 1st and 4th days immediately after fracture, because in our study the rats were sacrificed 6 weeks after fracture. We rather observed reparation and remodeling phases in our results. In the reparation phase, radiologically fibrous tissue and callus tissue surrounding chondrocytes are seen at the end of the 1st week and especially in the second week [17]. However, in the present study, bone formation was more prominent since the rats were sacrificed in a later period.

With demonstration of nervous innervation of the bone, the relationship between nervous system and bone remodeling has attracted more interest in recent years, and there are numerous clinical and experimental studies conducted on this issue [18–21]. Increased callus formation and rapid healing process have been shown after head trauma [19]. Although this healing process after central nervous system damage has not been fully clarified, increased callus formation has been reported to play an important role especially in the etiopathogenesis of heterotopic bone formation [18], whereas in the peripheral nervous system, peripheral nerve resections or neuropathies have showed negative effects on fracture healing. In a rat model designed by Aro et al., fibula fractures were found to not heal after removal of periosteal neural mechanoreceptors [20]. Both experimental and clinical evidence has shown the role of the autonomic nervous system in bone metabolism [18]. An association has been shown between leptin-dependent central control and bone cells [18]. A robust autonomic nervous system contributes to the maintenance of healthy bone tissue and fracture healing process. On the contrary, the autonomic nervous system disorders may cause an abnormal bone remodeling [18]. Beta adrenergic and neuropeptide receptors have been found in osteoblastic and osteoclastic cells. A direct neural and osteoclast cell communication via adrenergic receptors has also been demonstrated [18].

The sympathetic nervous system (SNS) is one of the main connections between the central nervous system and the skeleton, based on *β*-adrenergic receptor (*β*AR) signaling in bone cells. The stellate ganglion is an anatomic structure found in the cervicothoracic region, providing sympathetic innervation to the upper extremities, while increased sympathetic hyperactivity causes vasoconstriction and release of free oxygen radicals. This sympathetic hyperactivity also plays a role in the etiopathogenesis of these postfracture complications as in Sudeck's atrophy [18]. Based on the hypothesis that stellate ganglion block may be helpful in the suppression of this hyperactivity process, which is caused by the sympathetic nervous system, in prevention of vasoconstriction, and thus, in fracture healing, we performed excision of the stellate ganglion in our experimental group, and histopathological evaluation showed better healing in the experimental group, compared to the controls. The rate of predominantly immature bone and less cartilage formation was higher in the experimental group (72.73%) compared to the control group (25.00%).

In stellate ganglion block, a local blockage is produced in the nerves running to the upper extremities, no central nervous system damage is induced, and peripheral nerve integrity is maintained. In brief, denervation is not created, because denervation has been previously reported to have negative effects on healing. To our best knowledge, this is the first study conducted on this issue, and our results are original. However, we believed that our results should be supported by further studies with a larger sample size, different fracture models, and long-term follow-up results.

Selective block of the sympathetic trunk is applied in stellate ganglion block. Creating a complete sympathetic ganglion blockage is important to eliminate sympathetic effects and to obtain reliable study results. Therefore, in our experimental model, sympathetic blockage was induced by resection of the ganglion instead of an injection method. Namely, a complete blockage was created and applied as previously reported in the literature [22]. Again, forearm fracture models have been previously studied in rats and proven to be applied relatively easier [23]. In addition, we preferred the forearm fracture model since these fractures are commonly seen in humans.

Because there is no consensus among orthopedics in evaluation of fracture healing, Corrales et al. retrospectively reviewed 123 studies published between 1996 and 2006 [24]. In that study, the authors concluded that clinical+radiological criteria were used by 62%, radiological criteria alone by 37%, and clinical criteria alone by 1% of these studies. Despite their limitations, direct X-rays have a critical importance in evaluation of fracture healing [25]. Furthermore, different scales and indices have been developed in order to make direct radiographies a more objective, valid, and reliable method (e.g., maximum callus index). In our study, also direct X-rays were used for the evaluation of fracture line and callus formation, as suggested in the literature. In this study, evaluations were made for bone formation, union, and remodeling. In addition, these evaluations were performed by two separate orthopedists, and agreement between them was assessed. The onset of bone formation was higher in the experimental group compared to the control group both by the first observer (50% vs. 35%) and the second observer (40% vs. 30%), although the difference was not statistically significant. Again, orthopedists made different assessments in terms of callus formation and invisible fracture line, and a moderate agreement was found between them. This might have resulted from a lack of quantification and only qualitative evaluation made in our study. Also, because of the animal model design of the present study, lack of clinical evaluation might have affected our results. In addition, if an assessment would be made on human fractures, agreements of the orthopedists could be more different because of the different size and anatomical characteristics.

The study has some limitations. As a weakness of our study, different evaluations were made among the orthopedists with a moderate agreement. In addition, since the study was conducted on an animal model, our results could not be clinically assessed. On the other hand, our study is the first in the literature to demonstrate the effects of stellate ganglion block applied in the upper extremity, on blood supply to bone, density, vascularization, and bone metabolism, which is the strength of our study.

In conclusion, stellate ganglion block may be helpful in suppression of this hyperactivity process, which is caused by the sympathetic nervous system, in prevention of vasoconstriction, and thus in fracture healing. In our study, the rate of predominantly immature bone and less cartilage was significantly higher in the experimental group compared to the control group. Fracture healing was more prominent in the stellate ganglion group. There was a moderate agreement between the orthopedist in radiologic imaging made for the evaluation of fracture, union, and callus tissue; namely, the orthopedists drew different conclusions. We believe that further experimental studies are needed to fully clarify effects of stellate ganglion block on fracture healing.

## Figures and Tables

**Table 1:** Fracture region histologic findings between the experimental and control groups.

Fracture region histologic findings	Control		Experiment		*p*
	*n*	%	*n*	%	
Predominantly cartilage and less immature (woven) bone	6	(30.00)	1	(4.55)	0.006
Equally distributed cartilage and immature bone	9	(45.00)	5	(22.73)	
Predominantly immature bone and less cartilage	5	(25.00)	16	(72.73)	

**Table 2:** Comparison of bone formation, union, and remodeling results between the experimental and control groups by the first observer.

Observer 1		Control		Experiment		*p*
		*n*	%	*n*	%	
Bone formation	Callus formation	3	(15.00)	3	(13.64)	0.603
	Onset of bone union	7	(35.00)	11	(50.00)	
	Invisible fracture line	9	(45.00)	8	(36.36)	
	Complete bone union	1	(5.00)	0	(0.00)	

Union	No callus formation	1	(5.00)	2	(9.09)	0.259
	Callus formation	3	(15.00)	3	(13.64)	
	Onset of bone union	8	(40.00)	10	(45.45)	
	Invisible fracture line	4	(20.00)	7	(31.82)	
	Complete bone union	4	(20.00)	0	(0.00)	

Remodeling	No callus formation	3	(15.00)	4	(18.18)	0.573
	Callus formation	4	(20.00)	5	(22.73)	
	Onset of bone union	3	(15.00)	7	(31.82)	
	Invisible fracture line	6	(30.00)	3	(13.64)	
	Complete bone union	4	(20.00)	3	(13.64)	

**Table 3:** Comparison of bone formation, union, and remodeling results between the experimental and control groups by the second observer.

Observer 2		Control		Experiment		*p*
		*n*	%	*n*	%	
Bone formation	Callus formation	2	(10.00)	6	(27.27)	0.123
	Onset of bone union	6	(30.00)	9	(40.91)	
	Invisible fracture line	9	(45.00)	7	(31.82)	
	Complete bone union	3	(15.00)	0	(0.00)	

Union	No callus formation	1	(5.00)	1	(4.55)	0.408
	Callus formation	4	(20.00)	6	(27.27)	
	Onset of bone union	3	(15.00)	8	(36.36)	
	Invisible fracture line	6	(30.00)	3	(13.64)	
	Complete bone union	6	(30.00)	4	(18.18)	

Remodeling	No callus formation	3	(15.00)	5	(22.73)	0.679
	Callus formation	3	(15.00)	5	(22.73)	
	Onset of bone union	6	(30.00)	4	(18.18)	
	Invisible fracture line	3	(15.00)	5	(22.73)	
	Complete bone union	5	(25.00)	3	(13.64)	

## Data Availability

The data used to support the findings of this study are included within the article.
